# Effects of Physical Exercise on Cognition and Telomere Length in Healthy Older Women

**DOI:** 10.3390/brainsci11111417

**Published:** 2021-10-27

**Authors:** Juan Luis Sánchez-González, Juan Luis Sánchez-Rodríguez, Javier Martín-Vallejo, Abel Martel-Martel, Rogelio González-Sarmiento

**Affiliations:** 1 Department of Nursery and Physiotherapy, Faculty of Nursery and Physiotherapy, University of Salamanca, 37007 Salamanca, Spain; 2 Department of Basic Psychology, Psychobiology and Methodology, Faculty of Psychology, University of Salamanca, 37005 Salamanca, Spain; jlsanch@usal.es; 3 Department of Statistics, Faculty of Medicine, University of Salamanca, 37007 Salamanca, Spain; jmv@usal.es; 4 Department of Medicine, Faculty of Medicine, University of Salamanca, 37007 Salamanca, Spain; abelmartelmartel@usal.es (A.M.-M.); gonzalez@usal.es (R.G.-S.)

**Keywords:** exercise, cognition, adults, telomerase, ageing

## Abstract

Background: Physical exercise is an effective measure for preventing the onset of cognitive decline and has a direct influence on the aging process. The purpose of this study was to assess the effect of a 6-month physical exercise program on cognition and telomere length in adults over 65 years of age. Method: Seventy-four healthy women were separated into two groups: 41 were included in the intervention group (IG) (72.70 ± 4.127 years and 8.18 ± 1.551 years of education) and 33 in the control group (CG) (71.21 ± 4.127 years and 8.42 ± 2.562). The participants included within the IG carried out three sessions of physical exercise per week for six months. Cognitive function was assessed using the Mini-Mental State Examination (MMSE), the Stroop test and the Trail Making Test (TMT). Saliva samples were taken and analyzed and relative telomere length was calculated. Those conducting the analysis were blind to the group to which the participants had been assigned. Results: An improvement was observed in global cognitive function, in both attentional and executive functions, in the group of adults doing physical exercise as compared to the control group. Six months after the physical exercise program had finished, relative telomere length was found to have increased in the participants in the intervention group. Conclusion: Physical exercise programs can lead to an improvement in both cognitive functions and telomere length.

## 1. Introduction

New healthcare policies and advances in the field of health are leading to increased life expectancies and decreases in mortality rates, resulting in an inverted population pyramid (more elderly people). Consequently, this situation has given rise to a higher prevalence of chronic age-related diseases, which are associated with ageing populations.

Within this context, there are several theories that have attempted to account for the mechanisms of ageing that focus on telomerase and telomeres, important biomarkers of this natural process [1]. Telomeres are cellular structures composed of tandem DNA repeats (TTAGG) located at the end of chromosomes, and their main function is to protect chromosomes from degradation during each cell cycle [2]. Telomerase is the enzyme responsible for maintaining telomeric length (TL) by adding TTAGG repeats at 3’-ends during the retro transcription process [3]. TL shortening can be accelerated by factors that induce oxidative stress, carcinogenic processes, psychological disorders, cardiovascular disease, environmental pollution and exposure to tobacco smoke [4,5,6].

According to the literature, it has been estimated that telomere length in a population of males and females decreases by approximately 71–72 base pairs per year. Moreover, it has been observed that the short is more pronounced in males than in females. Thus, it takes longer to observe changes in relative TL in a female population than in a male population for individuals aged between 60 to 80 years [7].

It is known that moderate levels of physical exercise produce beneficial effects on cell regeneration and senescence by reducing telomere shortening and inducing a protective telomere phenotype, preventing age-related diseases [8]. Authors such Du and collaborators [9] have confirmed these results by concluding that moderate physical exercise is associated with good health and better survival; however, only a handful of studies have evaluated the effect after the end of the physical exercise programs.

The clinical evidence gathered over recent decades does indicate that older individuals (relative to other age groups) more frequently experience cognitive changes, with processing speed, attentional functions and executive functions being most affected during the aging process [10,11].

Subsequently, several authors have studied the consequences of physical exercise on brain structure [12] and have concluded that physical exercise produces less age-related atrophy in the prefrontal and temporal cortex [13], resulting in less decrease in cognitive function [14,15]. Some studies have also shown that an acute session of physical exercise improves cognitive performance, which may help explain the potential benefit of exercise on cognition [16,17,18]

The aim of our study, based on the effects that physical exercise has on TL and cognition, was to assess the impact of a physical exercise program (Geriatric Revitalization Program, (GRP)) on cognitive functions, such as attentional and executive, and the possible influence exercise may have on telomere length 6 months after the end of the GRP.

## 2. Materials and Methods

All participants signed an informed consent form and were informed of the details of the study, which had been approved by the Bioethics Committee of the University of Salamanca. Ethical standards and considerations were followed, as well as the protection of personal data and the privacy of the participants. Eleven participants received an identification number to guarantee their anonymity.

This is an experimental study including pre- and post-testing. For sample selection, a random sampling by clusters was carried out where eight neighborhood associations, out of a total of 20 older adults participating in the Geriatric Revitalization Program, were selected. Only those individuals from each association fulfilling all of the requirements were chosen to participate in the study. As a result, a total of 96 participants were selected. Of the total sample, 22 people abandoned the study for various reasons, reducing the sample size to 74 participants, 43 of which were assigned to the intervention group (IG) and 33 to the control group (CG). The inclusion criteria for the intervention group was the following: females participating in the Geriatric Revitalization Program aged between 65 and 80 years; non-smokers; not suffering from-diabetes, not taking antihypertensive medication; and not presenting any type of neurodegenerative pathology or neoplastic process. The inclusion criteria for the participants assigned to the control group were the same as those above, except the individuals did not take part in the Geriatric Revitalization Program or any other type of physical exercise program or activity. Therefore, the participants in the control group were described as being “currently inactive”, which was defined as not participating in regular physical activity or exercise of moderate intensity (30 cumulative minutes per day) on more than three days of the week, or exercising < 90 min/week [19,20]. The fact that the sample consisted only of women makes it possible to control those symptoms associated with the acceleration of telomeric shortening (already mentioned in the introduction section) such as smoking and alcohol since, according to epidemiological studies, women over 65 years of age exhibit fewer bad lifestyle habits which could influence telomeric activity than men.

The study period was between October 2018 and October 2019. In October 2018 (time point 0) the participants underwent a neuropsychological evaluation to assess global cognitive function, attention, executive functions and processing speed. Subsequently, those in the IG participated in a Geriatric Revitalization Program for 6 months, while the CG continued to carry out their daily lives as they had been doing prior to their involvement in the study. After 6 months (time point 1), the neuropsychological evaluation was repeated; however, at this time saliva samples were collected from all participants for quantifying telomere length (As mentioned in the introductory section, telomere shortening in women is slower than in men. Our study sample is only formed by women so we started from the hypothesis that in 6 months we were not going to appreciate significant changes in the length of their telomeres). During the next 6 months, all participants (in both the IG and the CG) were described as being “currently inactive”. To control this situation, telephone contacts were maintained every 2–3 weeks. At the end of this 6-month period (time point 2) another saliva sample was taken and again telomere length was quantified.

In addition, due to the possible influence of weight, fat weight and fat percentage on telomere length [21,22], these parameters were measured before and after the intervention in the subjects of both groups in order to control the changes produced in these variables.

The tests employed for assessing the cognitive functions were the following: the Mini Mental State Examination for evaluating global cognitive function [23]; the Stroop Test for attentional and executive functions [24,25]. The scoring method of the Stroop Test has been obtained, taking into account two fundamental requirements: On the one hand, the accuracy has been calculated for each of the test conditions (Words, Colors and Words/Colors) [26,27]. On the other hand, the global index has been calculated to relate the performance in the incongruent condition to reading words and color naming abilities. The calculation of accuracy was performed by counting the number of correct responses in each condition within a fixed time (45 s). The global index was calculated according to the formula:

Global Index = CW − [(W × C)/(W + C)]

where: CW: number of items properly named in 45 s in the CW condition; W: number of items properly named in 45 s in the W condition; C: number of items properly named in 45 s in the C condition.

The latest tests used were parts A and B of the Trail Making Test for processing speed [28].

DNA was extracted from saliva using the following protocol [29,30]. The cells were isolated by centrifugation and resuspended in Fornace buffer (0.25 M sucrose, 50 mM Tris-HCl pH 7.5, 25 mM KCl, 5 mM MgCl_2_) followed by an additional centrifugation at 1500 rpm for 10 min. The resulting pellet was incubated at 55 °C for 8–16 h in Fornace buffer containing 10 mM EDTA pH 8.0, 1% SDS and proteinase K (ApliChem, Castellar del Vallès, Spain) for protein degradation and for breaking down the cell membrane. Then, DNA was extracted and purified using the phenol-chloroform method and precipitated using cold absolute ethanol.

The concentration of the extracted DNA was determined by measuring the absorbance at 260 nm using a NanoDropTM 2000/2001 spectrophotometer. The purity of the DNA was analyzed based on the A260/280 absorbency ratio, where an optimal purity ratio ranged between 1.8–2.0. All DNA samples were stored in Eppendorf tubes at −20 °C.

The telomere length of the saliva cells taken from each participant was measured using quantitative real-time PCR (qPCR) together with the Absolute Human Telomere Length Quantification qPCR Assay Kit (ScienCell, Catalog #8918, Faraday Ave, Carlsbad, CA, USA).

This technique allows the initial amount of DNA coding for telomerase (TEL) to be quantified and compared with that obtained simultaneously from another fragment corresponding to a single copy reference gene (SCR) exerting endogenous control. The difference in the amount of DNA quantified represents the relative TL of each participant. In order to analyze these relative changes, a reference fragment (CONTROL) of known TL (provided by the manufacturer) was added to each assay, allowing the absolute quantification of the telomere length of each sample.

The reactions took place in a Micro-Amp Fast Optical 96-Well reaction plate (Applied Bio systems) and the TEL and SCR fragments from the DNA samples of participants were amplified using the Applied Biosystems StepOnePlusTM Real-Time PCR System. Triplicate reactions were carried for each sample to minimize variability. The TEL and SCR fragments were amplified using 1 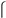
L of each specific primer, 10 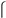
L of the FastStart SYBR Green MasterMix and 7 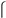
L of water. The total amount of DNA used for each reaction was 10 ng in 2 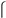
L. The amplification program was as follows: 10 min at 95 °C, followed by 40 cycles at 95 °C for 15 s, 52 °C for 30 s and 60 °C for 1 min.

Finally, the Ct (2^−ΔΔCt^) comparative method was used to calculate the relative expression levels of each amplicon. The specificity of each PCR was checked by verifying the TL length of the reference sample (CONTROL), which in turn allowed the absolute lengths of each sample for a diploid cell and/or chromosome end to be determined. The program Excel was used for the management of data. The procedures used in this study were approved by the Bioethics Committee of the University of Salamanca and were therefore carried out in accordance with the ethical standards set out in the Declaration of Helsinki 1964.

In this study, the participants in the IG were asked to carry out 3 sessions of physical exercise per week for 6 months as recommended by the World Health Organization [31]. As established by the Geriatric Revitalization Program, each session consisted of 3 main parts: a dynamic warm-up involving the mobility of the main joints and aerobic exercise (10 min of jogging); an extended period of strength-resistance exercise for the main muscle groups (chest, dorsum, biceps, triceps, deltoid, shoulders, quadriceps, gluteus, biceps femoral, rectus femoral, gastrocnemius, abdominal) of the lower and upper extremities (once this part had ended the participants were allowed to drink water and rest and attendance was checked so as to keep strict control over participation); and a final phase of relaxation and a period of calm involving stretching the muscle groups that had been worked on during the session. A sample session of the Geriatric Revitalization Program is shown in List S1 and Figure S1 in the Supplementary Materials.

Body composition was evaluated using the OMRON BF300 monitor and weight, height, fat weight and body fat percentage were determined.

Means, standard deviations, medians and interquartile ranges were used to describe the data obtained for the qualitative variables, which were calculated as percentages. The analysis of the normality of observations was carried out by means of the Lilliefors-corrected Kolmogorov test.

When comparing the two groups, the *t* test was used for the independent data, if the variables followed normal distributions and the non-parametric Mann–Whitney U test in the case of non-normal distributions. For choosing the degrees of freedom for the *t* test, the Levene’s test was used to test the equality of variances. The reliability of measures in the different scales of the STROOP test were intraclass correlation.

The two-factor ANOVA was used to analyze the change over time in cognitive function between the intervention and control groups, one of the factors being the independent measures (IG/CG) and the other the dependent measures (pre and post). In cases in which the instrument had several dimensions, the three-factor ANOVA was used; one of independent measures (IG/CG) and two of dependent measures, being time (pre and post) and dimension. A linear mixed model was used to analyze the change in telomeric length. Given the importance of the influence of weight and body fat on telomere length, changes in weight and the percentage of fat between intervention periods were included in the model as covariables. Thus, the effect of these uncontrolled variables is removed on the dependent variable and so the means of the intervention groups have been adjusted by these covariables The proposed linear mixed model was:

(1) $$TL=b_{0}+b_{1}*IG_{ij}+b_{2}*WC_{ij}+b_{3}*\%FAT_{ij}+b_{4}*T_{ij}+b_{5}*IG_{ij}*T_{ij}+r_{j}+e_{ij}$$

where:

*TL* = Telomeric Length; *IG* = Intervention group; *WC* = weigh change; %*FAT* = *FAT* percentage; *T* = time; *r_j_* is the random effect and its probability distribution: N (0; σ) and *e_ij_* is the error term. i = 1,74; r = 1,2

The interaction effects are shown by interaction graphs. Bonferroni correction was applied to multiple comparisons. The significance level 0.05 was chosen. IBM-SPSS v.26 was used to analyze the data and all data were analyzed at the end of 2019.

## 3. Results

A descriptive analysis of the participants’ socio-demographic variables was carried out to examine the comparability of the sample groups. The descriptive data of the sociodemographic and anthropometric variables are presented in Table 1.

For the rest of the variables studied, we were interested in ascertaining if any changes had taken place between the IG and the CG.

First, with regard to global cognitive function, the results indicated that none of the participants presented global cognitive deterioration, based on the fact that the average scores obtained were in the normal range (Figure S2). Secondly, it was detected that the interaction between the intervention and the time points was significant (*p*-value < 0.0001); that is to say, the change that took place during time point 1 in the IG was different from that found in the CG.

With regard to the attentional and executive functions (Table 2), the three factor interaction was not significant (*p*-value = 0.499) among the time points and both groups. The only significant interaction was associated with the time points and the IG (*p*-value < 0.0001), where the change observed between time points 0 and 1, for the IG, was statistically significant. In addition, these differences were maintained for the different dimensions of the Stroop Test. When the scores of the different dimensions of the Stroop Test were analyzed in detail, it was found that the participants in the CG, at time point 0, presented higher values. However, at time point 1, the results were the opposite and the IG scored slightly higher for all the dimensions analyzed. The intraclass correlation values for Word reading, Color naming, Word/Color and Naming with interference were 0.70, 0.58, 0.52, 0,48, respectively. The values of the global index and accuracy of each of the dimensions (Words, Colors and Words/Colors) of the Stroop Test are shown in Table S1 of the Supplementary Materials. These values have been corrected for age.

Regarding parts A and B of the Trail Making Test, the three-factor interaction effect was statistically significant (*p*-value = 0.002). This interaction indicates that the participants in the IG performed the trace test, both in part A and B, more quickly at time 1 (intervention time) than time 0 (basal time). However, the participants of the CG performed the test more slowly at time 1 than time 0, but the magnitude of the differences was significantly larger in part A than in part B.

**Figure 1:**
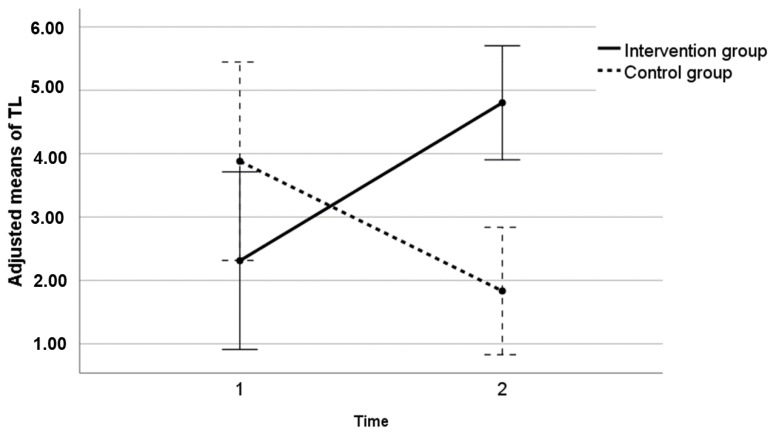
Graph representing the interaction between the experimental groups and time with respect to TL. The points represent the adjusted means by weight change and % fat change. The 95% confidence intervals for the adjusted means are also represented. Time 1: telomere length values at the end of the intervention in both groups. Time 2: telomere length values 6 months after completion of the intervention.

**Figure 2:**
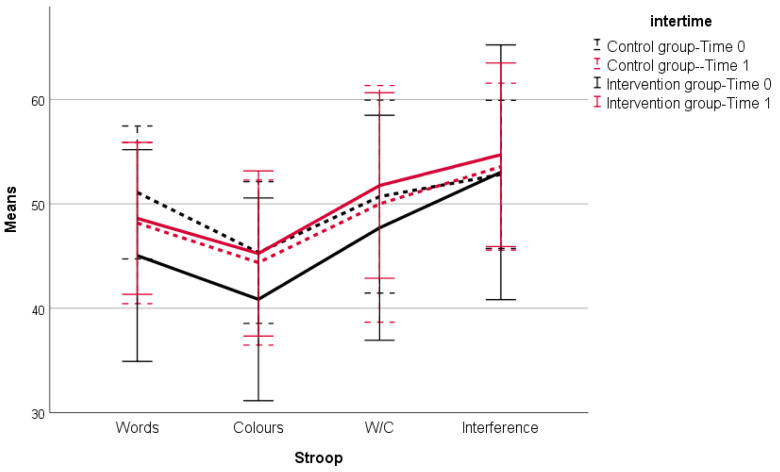
Interaction between both intervention groups at time point 0 and time point 1. Mean ± Standard Deviation: scores obtained by the subjects in each of the Stroop Test dimensions (Words, Colors, Words/Colors, Interference) before and after the intervention. Words: number of items properly in 45 s in the Words condition; Colors: number of items properly named in 45 s in the Colors condition; W/C: number of items properly named in 45 s in the Words/Colors condition; Interference: Naming with interference.

The means and standard deviations of TL in CG and IG were 3.88 ± 6.030 and 2.30 ± 2.413 at time point 1, respectively. The descriptive values of TL during the second time point were 1.81 ± 2.34 in CG and 4.81 ± 3.16 in IG (Figure 1). With regard to the results obtained for telomeric length, a statistically significant difference was detected in the interaction between both groups and time points 1 and 2. In other words, the change of TL that occurred between each time point was different for the IG compared to CG (*p*-value < 0.001). As can be observed in Figure 2, the IG shows an increase in TL at time point 2, after the intervention, while a decrease in TL can be observed for the CG. It can also be seen that the difference between the two groups at time point 1 is smaller than at time point 2, and that the results are inverted. It may be noted that the means have been adjusted by weight and percentage fat to avoid the interference of these uncontrolled variables with the effect of intervention in telomeric length.

## 4. Discussion

Currently, the ageing of the world’s population has major social and economic implications. Thus, more than ever, it is necessary to emphasize strategies that lessen the clinical manifestations of age-related diseases. The aim of this research was to analyze the effects that the Geriatric Revitalization Program (GRP) could have on aging, specifically on cognitive functions and telomere length. As mentioned in the methods section, the GRP is a physical exercise program that combines aerobic with resistance-strength activities.

This work is based on the assumption that the participants of the IG would exhibit, after the intervention, an improvement in their global cognitive function, attentional and executive functions and processing speed. It was also thought they would obtain higher scores with respect to those participants included within the CG.

With respect to the Mini Mental State Examination, the results show a positive interaction with regard to the scores obtained by both groups for the two measurements, i.e., the participants in the IG obtained significantly higher scores on the MMSE after partaking in the Geriatric Revitalization Program. Authors, such as John and collaborators [6], obtained results identical to those obtained in this work for a sample of 199 individuals after 12 months of intervention. However, it should be noted that in our study an improvement was observed in the IG after only 6 months. Lin and collaborators [32] also obtained results similar to ours when studying a sample of 2074 people. They divided the sample into two groups: an intervention group (1372) that engaged in physical exercise and a control group (702) that did not. These authors found that those in the intervention group showed better cognitive performance on the MMSE as compared to the participants in the control group. In addition, those in the intervention group obtained better test scores after the intervention, as was the case with the participants in our study.

As for the executive functions, in particular attentional processes, the results follow a similar trend, as previously mentioned; the participants in the IG show an improvement with regard to these variables after practicing physical exercise. In a meta-analysis [33], including 29 randomized clinical trials, it was concluded that physical exercise leads to enhancements in the executive functions, such as processing speed and attention, findings that are in line with our results. Cross-sectional studies have also shown that physical exercise has a positive effect on executive functions. Bixby and collaborators [25] have also shown that physical exercise is associated with the improved performance of executive functions after studying its effect in a sample of 122 people between the ages of 68 and 92. As mentioned above, the IG in our study scored better in relation to processing speed, where a significant improvement was observed after the intervention. These results are in line with those of other authors, such as Spartano and collaborators [34], who studied a cohort of 909 older adults. They concluded that subjects with higher levels of physical exercise have enhanced executive functions. Moreover, Albinet and collaborators [35] observed an improvement in the executive functions of the women in an intervention group, as compared to the controls, after a 5-month physical exercise program.

Lastly, in relation to the Trail Making Test, the participants of the IG performed the tracing task in less time than the participants of the CG after the intervention. This indicates that there is a significant improvement in perceptual-motor speed, processing speed, visual attention and flexibility. These results are in line with those published by Mekari and collaborators [36] who observed an association between the level of cardiorespiratory fitness and cognitive performance, specifically for task B of the Trace Test (perceptual-motor speed). They showed that out of the 66 people participating in the study, of which 44 were women, those who presented greater cardiorespiratory fitness also performed better on this task. The explanation for these results was that the positive relationship between cardiorespiratory fitness and perceptual-motor speed was mediated by better brain oxygenation; that is, older adults with greater brain oxygenation are able to respond to more demanding tasks. Cross-sectional studies [37] are also in line with our results, which show that older adults with increased cardiorespiratory fitness perform better on tasks involving perceptual-motor speed.

With respect to the second hypothesis proposed in our study, we assumed that the participants in the IG would have greater telomere length at the end of GRP, as compared to the CG, and would continue to show an increase in TL 6 months after the program had been completed. 

Our results indicate that at time point 1 those in the CG had a greater TL than the participants of the IG. This could be due to the dramatic impact that physical exercise has on the health of individuals, causing an increase in the reactive oxygen species and, as a result, a decrease in TL [38,39]. However, 6 months after the GRP had ended, the adults in the IG presented an increase in telomeric length, while the CG showed a decrease. These results imply that the impact of physical exercise on TL does not occur immediately, but rather in the medium to long term by reducing the levels of reactive oxygen species. A recent study [40] showed that in a sample of 1481 elderly women, those who practiced moderate physical exercise had greater telomere length.

However, in recently published reports there is some controversy regarding the impact of physical exercise on TL. Some focusing on aerobic exercise, such as work by Puterman and collaborators, have concluded that a 45-min aerobic exercise program 3 times per week caused an increase in telomere length in a group of 68 people between the age of 50 and 75 years. However, there are other studies with contradictory results regarding the impact of this type of exercise on TL [19,41].

In addition, there are studies that focus mainly on resistance exercise gain with contradictory results. Some research has shown that endurance training causes adaptive changes in the antioxidant protein systems at the systemic level leading to a maintenance of TL [42], while other studies have concluded there is no effect at all [43,44] or even a shortening of TL [45]. Nevertheless, in this work we observed that in those subjects who had participated in a program involving both types of exercise (Aerobic exercise + Resistance exercise) there was an increase in TL 6 months after the program had ended. The great variability in the application of physical exercise programs, the different exercise modalities and the high heterogeneity of the samples studied could be one of the possible reasons for the contradictory results.

Furthermore, none of the abovementioned studies have examined what happens after the end of a physical exercise program. To date, we have found only one article that assesses the impact of a resistance exercise program 12 months after the end of the program. Nickels et al. [46] conclude that those subjects who returned to a sedentary lifestyle during the 12 months after the intervention showed a significant reduction in telomere length. However, those subjects who continued to exercise after the intervention did not show reduced telomere length. These results are similar to those obtained by us for the control group, as they continued to lead a sedentary lifestyle and showed a decrease in their telomere size. However, the subjects in our study who participated in an aerobic exercise and resistance exercise program 6 months after the end of the program not only did not wear out their TL but showed a significant increase in their TL.

Another important observation detected in our study is the fluctuation that exists in telomeric length in older adults with or without intervention during a relatively short period of time. In addition, we have shown that telomeric fluctuation does not follow a linear process, but instead is dynamic. Furthermore, Svenson and collaborators [47] showed after studying a sample of 50 people, most of whom were women, that during a 6-month follow-up period telomere length could become shorter and longer throughout this period. Thus, telomere length could be considered a dynamic characteristic which is influenced by physical exercise.

Finally, one of the main limitations of this work could be that the changes induced by exercise could have been influenced by psychosocial factors associated with the social interactions experienced by the participants when exercising in a group setting since there are studies that claim that people with depression and/or anxiety present a shorter telomere length [48,49]. Changes in these psychosocial factors could influence telomere length. Furthermore, TL was not assessed at time 0 but at the end of the intervention and at 6 months after the intervention in order to observe what happened after the end of the physical exercise program.

In addition, the sample of our study was composed only of women, so the results found should not be generalized to the general population.

Therefore, further studies with larger and more heterogeneous samples and taking into account social factors and interactions are needed.

## 5. Conclusions

Physical exercise programs, such as the Geriatric Revitalization Program, can be a useful strategy for slowing down the loss of age-related cognitive functions and for slowing down aging in general. The implementation of this type of program by health professionals could help prevent age-related diseases or deficiencies.

However, the groups involved in the present study were composed of a small sample size, and the data should be interpreted with caution.

## Figures and Tables

**Table 1:** Descriptive statistics by age group and anthropometric variables.

Statistical Descriptions	Control Group N = 33	Experimental Group N = 41	*p*-Value
Age (years)	Mean (SD)	Mean (SD)	0.138
	71.21 (4.329)	72.70 (4.13)	
Weight (kg)	Mean (SD)	Mean (SD)	
Pre	62.72 (8.49)	64.98 (8.41)	0.258
Post	63.13 (8.40)	64.70 (8.85)	0.438
%Fat (%)	Mean (SD)	Mean (SD)	
Pre	38.73 (5.52)	43.07 (3.60)	<0.001
Post	39.37 (5.45)	42. 81 (4.93)	0.006

Note. SD = Standard Deviation.

**Table 2:** Mean, Standard deviation and *p* value for the Mental State Examination, the Stroop Test and the Trail Making Test over time (pre and post) for the control and intervention groups (CG/IG).

Variables	Time 0		Time 1		*p* Value
	Control	Intervention	Control	Intervention	
	Mean (SD)	Mean (SD)	Mean (SD)	Mean (SD)	
Mental Status					
MMSE (max of 30)	28.64 (1.50)	27.67 (1.72)	27.64 (1.75)	28.40 (1.50)	<0.0001
Attention					
Stroop Color–Word Test (number of items properly named in 45 s)					
Word reading	50.78 (6.21)	45.40 (10.02)	48.16 (7.72)	48.50 (7.34)	<0.0001 *
Color naming	45.22 (6.86)	40.70 (9.78)	44.38 (7.91)	45.20 (8.01)	0.015 *
Word/Color	50.97 (9.25)	47.32 (10.63)	50.00 (11.34)	51.63 (8.96)	0.200 *
Naming with interference	53.31 (6.61)	52.55 (11.97)	53.56 (8.00)	54.70 (8.79)	>0.999 *
Executive Functions					
Trail Making A (Seconds)	56.19 (25.63)	81.68 (35.74)	67.25 (28.95)	63.80 (22.46)	<0.001 *
Trail Making B (Seconds)	151.38 (85.23)	217.02 (108.52)	178.44 (106.75)	171.22 (79.02)	<0.001 *

Note. SD = Standard Deviation. * Bonferroni adjustment. MMSE = Mini Mental State Examination.

## Data Availability

The datasets used and/or analyzed during the current study are available from the corresponding author on reasonable request.

## Supplementary Materials

The following are available online at https://www.mdpi.com/article/10.3390/brainsci11111417/s1, Figure S1: Main part and period calm, Figure S2: Interaction of both groups with respect to the MMSE, Table S1: Mean scores of the global index and accuracy of the age-corrected dimensions of the Stroop Test, List S1: Dinamic warm-up.
