# Supplementary material for: Effects of Physical Exercise on Cognition and Telomere Length in Healthy Older Women
Source: Brain Sci. 2021 Oct 27;11(11):1417. doi: 10.3390/brainsci11111417 (PMC8615568; doi:10.3390/brainsci11111417)
Supplement: Supplementary file 1 [file brainsci-11-01417-s001.zip › brainsci-1429034-supplementary.pdf]

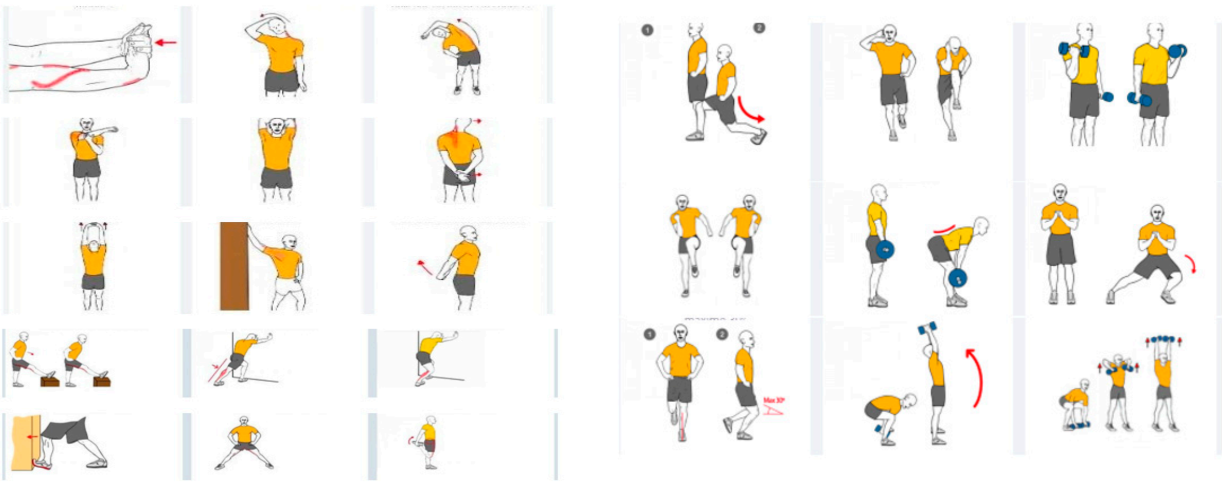

(**A**) (**B**)

Figure S1. A: Main part: Between 10 and 15 repetitions each exercise with a rest of 1 minute between each exercise. **B:** Period of calm: Stretching of the main muscle groups.

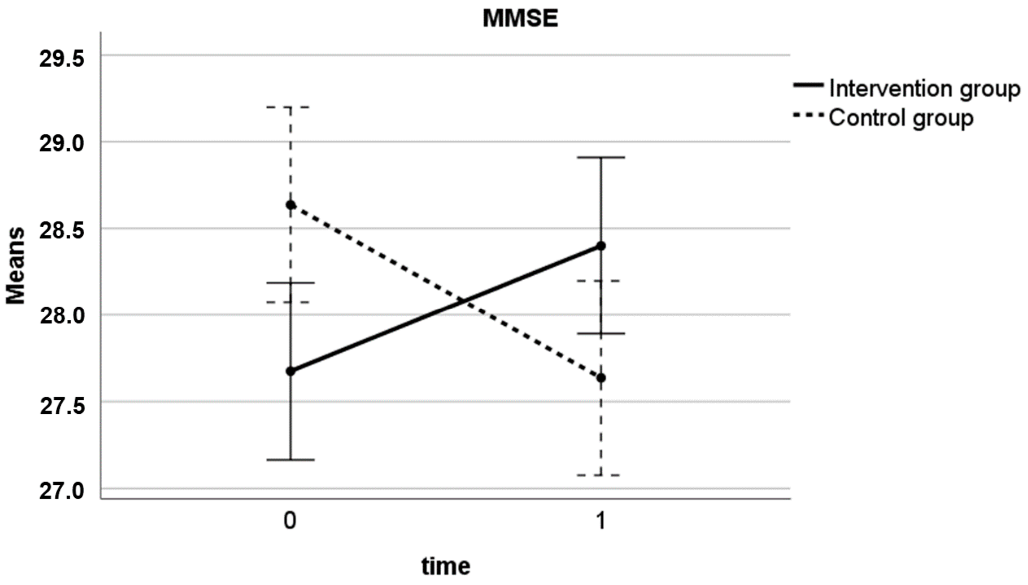

Figure S2. Interaction of both groups with respect to the MMSE. Mean ± Standar Deviation_ Time 0: scores obtained by the subjects in the Mini Mental State Examination before the intervention. Time 1: scores obtained by the subjects in the Mini Mental State Examination just after the intervention.

Table S1. Mean scores of the global index and accuracy of the age-corrected dimensions of the Stroop Test.

		Time 0		Time 1	
**Dimension**	**Groups**	**Mean**	**SD**	**Mean**	**SD**
Words	Intervention	45.40	10.02	48.50	7.34
	Control	50.78	6.21	48.16	7.72
Colors	Intervention	40.70	9.78	45.20	8.01
	Control	45.22	6.86	44.38	7.91
Words/Colors	Intervention	47.33	10.63	51.63	8.96
	Control	50.97	9.25	50.00	11.33
Global Index	Intervention	52.55	11.97	54.70	8.79
	Control	53.31	6.61	53.56	8.00

Note. SD = Standar Deviation. Words = number of items properly in 45 seconds in the Words Condition; Colors = number of items properly named in 45 seconds in the Colors Condition; Words/Colors: number of items properly named in 45 seconds in the Words/Colors Condition. Global index or Interference Values have been adjusted for age.

Supplementary List S1

Dinamic warm-up

Mobility of the main joints:

- Opening and closing the hands
- Circumduction of wrists
- Elbow flexion-extension
- Circumduction of shoulders
- Bringing the arms together and spreading them apart
- Raise the shoulders
- Alternately raise your arms in extension until they are in line with your head.
- Rotation of the trunk in the vertical axis
- Lateral flexion of the trunk in the anteroposterior axis
- Flexion of the trunk in the transverse axis
- Circumduction of the hip
- Semitandem leg flexion, with trunk flexion
- Arm in extension above the hip, with trunk flexion touching the opposite knee.
- Skipping or hip flexion to =<90º.
- Lateral hip flexion up to =<90º (open leg)
- Total knee flexion (heels to butt)
- Circumduction of knees
- Walking backwards
- Lateral walk
- Crossed front and back walk
- Circumduction of ankles
- 10 minutes of jogging
